# ‘It made you think twice’ – an interview study of women’s perception of a web-based decision aid concerning screening and diagnostic testing for fetal anomalies

**DOI:** 10.1186/s12884-016-1057-y

**Published:** 2016-09-13

**Authors:** Annika Åhman, Anna Sarkadi, Peter Lindgren, Christine Rubertsson

**Affiliations:** Department of Women’s and Children’s Health, Uppsala University, Box 609, Uppsala, 751 25 Sweden

**Keywords:** Decision aid, Genetic screening, Informed choice, Pregnancy ultrasound, Internet

## Abstract

**Background:**

Enabling women to make informed decisions is a key objective in the guidelines governing prenatal screening and diagnostics. Despite efforts to provide information, research shows that women’s choice of prenatal screening is often not based on informed decisions. The aim of this study was to investigate pregnant women’s perceptions of the use of an interactive web-based DA, developed to initiate a process of reflection and deliberate decision-making concerning screening and testing for fetal anomalies.

**Methods:**

A qualitative study was applied and individual interviews were conducted. Seventeen pregnant women attending antenatal healthcare in Uppsala County, Sweden, who had access to the decision aid were interviewed. Eleven opted to use the decision aid and six did not. Data were analysed by systematic text condensation.

**Results:**

Women appreciated the decision aid, as it was easily accessible; moreover, they emphasised the importance of a reliable source. It helped them to clarify their own standpoints and engaged their partner in the decision-making process. Women described the decision aid as enhancing their awareness that participating in prenatal screening and diagnostics was a conscious choice. Those who chose not to use the web-based decision aid when offered reported that they already had sufficient knowledge.

**Conclusions:**

The decision aid was able to initiate a process of deliberate decision-making in pregnant women as a result of their interaction with the tool. Access to a web-based decision aid tool can be valuable to expectant parents in making quality decisions regarding screening for fetal anomalies.

## Background

Pregnancy is associated with great hope and high expectations but may also include fear that something might go wrong. In our modern society, information is all around us and readily available for expectant parents having any questions or concerns. As a consequence, the concept of pregnancy as a natural state [1], is competing with the diametrically opposed discourse of pregnancy as being a state of risk [2], where medical technology is suggested to be one way of gaining control [3]. Prenatal diagnostics and screening for fetal anomalies is such a technique that is widely available for women in developed countries and it is fast becoming ingrained in obstetric care also in developing countries [4]. Therefore, screening and diagnostic testing for fetal anomalies becomes a central part of many expectant parents’ realities.

Enabling women to make informed decisions is a key objective in the guidelines governing prenatal screening and diagnostics. This implies a decision that is based on relevant knowledge, is consistent with the decision-maker’s own values, and the ability of the person to act accordingly [5]. Despite efforts to provide information, research shows that women’s participation in prenatal diagnostics is often not an informed decision [6]. Moreover, a Swedish study concluded that counselling regarding prenatal testing in maternal healthcare does not fulfil the requirements needed to support expectant parents’ informed choice [7], suggested to be due to lack of time or level of counselling skills. It has also been shown that not all expectant parents read the informational pamphlets they receive from the midwife [8, 9]. Consequently, there is significant risk that women undergoing routine prenatal screening and testing are making uninformed choices due to their lack of active reflection and deliberate decision-making regarding their participation.

In Sweden, all pregnant women are offered a mid-trimester ultrasound examination, including screening for fetal anomalies as part of the antenatal healthcare services, most often performed by specially trained midwives. Only two per cent of the Swedish women refrain from this examination [10]. It is free of charge, and research suggests that parents perceive this ultrasound screening more as a social event rather than a medical examination [11]. First trimester screening for Down syndrome, the combined ultrasound and biochemical profile (CUB), is also offered at most fetal medicine units but in some counties only to women above 35 years of age [12]. Therefore, the uptake varies between counties, and on the national level the uptake was 36% in 2013. Since the introduction of the CUB-test, invasive diagnostic testing by amniocentesis (AMC) has decreased with an uptake of about two per cent in 2013, while chorionic villus sampling (CVS) was performed in one per cent of the pregnant women [10]. However, regional differences exist in terms of uptake, costs and to which groups of women (>35 years or all women) these procedures are offered.

Midwives are the main providers of antenatal care in Sweden and thus responsible for informing women about prenatal examinations [13]. However, the vast majority of pregnant women also seek and get pregnancy-related information from the Internet. A Swedish study based on data from 2004 shows that 84 % did so at a time when 91 % had Internet access [14]. In 2012, close to 100 % of women between 25–34 years had access to the Internet in their homes, implying that even more women access pregnancy-related information through the Internet today [15]. Even Swedish men seek pregnancy-related information on the Internet, although not to the same extent as women [16].

Use of a decision aid (DA) before a screening or specific treatment in healthcare is known to increase the likelihood that people will make decisions based on informed choices [17]. This is also reported for prenatal diagnosis [18]. The goals of decision aids are to help individuals make specific and carefully considered choices that are aligned with their own values and to explain the risks, benefits and consequences of available choices regarding tests or treatments in healthcare [19].

Decision aids, in general, include a section with information about various risks with different methods, benefits and consequences, and likelihood of certain results based on the individual’s *a priori* risk. They also include a worksheet with specific exercises to clarify one’s own values as well as act as a guide in decision-making. Different interactive decision support tools concerning screening and diagnostic testing for fetal anomalies have shown to result in more informed decisions than standard education booklets [19, 20].

Although the use of patient DAs has been studied in different contexts, including the area of fetal diagnostics [21], qualitative research describing users’ perspective and the processes that characterise patients’ interactions with DAs is still limited.

### Aim of the study

The aim of this study was to investigate pregnant women’s perceptions of the use of an interactive web-based DA, developed to initiate a process of reflection and deliberate decision-making concerning screening and testing for fetal anomalies.

## Methods

This qualitative study was embedded in a randomised controlled trial (RCT) that was initiated to evaluate a web-based DA developed for this study. The primary outcome in the RCT was informed choice according to Marteau et al [5]. The intervention group received access to the DA as a complement to standard care, i.e. oral and written information provided by antenatal care midwives. Participants were initially recruited for the RCT by antenatal care midwives in the catchment area of Uppsala University hospital between January and August 2013, and consented to participate. Inclusion criteria were: having a personal e-mail address and Internet access, being fluent in Swedish, wanting information about screening and diagnostic testing for fetal anomalies and being randomised to the intervention with the DA. Women got access to the DA via a website through a personal login.

In total, a purposive sample of 24 women was contacted during a two month period 4–8 weeks after their second trimester routine ultrasound screening and asked a) if they had used the DA and b) if they wanted to participate in an interview about their experiences. This time point was chosen in order to allow for any diagnostic procedures to be completed. The first six women, who were invited to participate in an interview, received the invitation in connection with the second questionnaire of the RCT, which was sent through e-mails. As these women all declined participation, we opted for contact by telephone 3 to 5 weeks after they answered the questionnaire. Of the 18 women who were then contacted by telephone, only one declined for medical reasons. Eleven of the 17 women who agreed to participate had used the DA and were invited to a face-to-face interview, which ten women accepted, whereas one preferred a telephone interview. In addition, short telephone interviews were conducted with six of the women who had not used the DA. The purpose of this was to get a sense of possible issues that might hinder expectant parents to use the DA that they had access to. The study was approved by the Regional Ethics Committee Uppsala, Dnr 2012/062. Written consent was obtained from participants at the time of the interview.

### Development of the web-based DA

The web-based DA designed for this project was based on a DA previously used in an Australian study [19]. It was built on the Ottawa decision support framework [22], and consideration was given to the quality criteria for decision support in healthcare described in the International Patient Decision Aid Standard (IPDAS) [23].

Our research team, having several years of experience in prenatal diagnostics, produced the DA website in collaboration with a qualified web designer and the Fetal Medicine Unit of the University Hospital. A genetic counsellor reviewed the DA, and a pilot test was performed on a paper version with three pregnant women. The online version of the DA was also pilot tested with four pregnant women. The results of the pilot test were used to make some minor changes in both layout and how the information was presented.

This DA is divided into four main modules: two modules focusing on facts, a third module with narratives providing illustrative examples of others’ experiences and actions relevant to the decision [24], and a fourth module with interactive ‘worksheets’ aiming to clarify one’s own values (Table 1). A link to the Fetal Medicine Unit’s homepage was inserted in connection with the information about available tests. To minimise potential bias in the presentation of the material on the website, neutral colours such as black, white, grey and light blue were used, and only a few pictures were included, illustrating the technical device used for the different screening tests.

**Table 1:** The different components of the decision aid with headings, sub headings and short descriptions of the content

Heading	Sub heading	Content
1. About fetal screening and diagnostics	Facts	• What tests are offered • What time in pregnancy the different tests are performed • What information the tests can provide, with separate pop-up windows that explain concepts such as ‘ultrasonographic soft markers’ and ‘the most common trisomies’ • Any risks associated with the tests and what the costs are • An Internet link to the official website of the fetal medicine unit at Uppsala University Hospital
2. Likelihood of anomalies	What do the results mean?	• Examples on how the risk figure 1 in 200 can be described in words and images • A chart that shows the proportion of DS fetuses related to women’s age • Information and examples on what clinicians express as high and low risk for DS, and what that equals in real figures • A prompt to the user of the DA to assess the chance of the fetus not having DS at the women’s own age
3. Expectant parents’ stories	Four couple’s choices	• Four different fictional stories in 150–200 words on how the couples think, their choices, and possible outcome of their choices
4. Worksheets	What is valuable to you?	• Sets of statements regarding personal opinions on what high or low risk for DS means, and their value of receiving information on one’s personal risk for carrying a fetus with DS • Series of claims on personal values that users are requested to take a stand on to promote awareness of one’s own values, using Ottawa decision weight scale [36]

### Setting

This study was conducted in Uppsala County where all antenatal centres refer women for fetal screening to the University Hospital in Uppsala. Approximately 4,000 women attend mid-trimester ultrasound screening each year, and about 25 % of these women also choose the CUB-test. In Uppsala County, the CUB-test has been offered primarily to women above 34 years of age at a subsidised cost of 300 Swedish Crowns (SEK), while younger women had to pay the full costs for the examination which was 1,500 SEK at the time of this study. Following the CUB-test, invasive tests are offered to women at ‘high risk’ for fetal trisomies, which in this setting was a risk score of ≥1/200. Information on available screening procedures for fetal anomalies is provided orally, in combination with a locally developed pamphlet, and if needed, via a consulting obstetrician.

Semi-structured interviews were conducted at a location of the women’s preference, most often at the researcher’s office. Gestational age at the time of the interview was between 24 and 30 weeks.

The interviews with the users of the DA, lasting 20 to 43 min (median 30 min), were conducted while sitting in front of the computer, using the website as a guide together with an interview guide with questions focusing on participants’ utility of the DA and user friendliness (Table 2). The telephone interviews with non-users, which lasted 3 to 5 min, were focused solely on their personal reasons for not using the DA. All interviews were recorded with the consent of the participants and transcribed verbatim.

**Table 2:** Interview questions for users of the decisional aid and for non-users

Main question to users	Probing questions
While watching the website, can you tell me how you perceived the different parts and what your thoughts were when using this tool?	- How did you perceive this part? - How did you perceive the features of this part of the DA? - Is there any information here that you did not receive elsewhere? - Do you have any suggestions on how to improve the design of this website? - How did it affect you? - What significance did the tool have for you?
Main question to non-users	Probing questions
Why did you not use the decision aid?	- From where did you receive information about the tests/ ultrasound examination?

**Table 3:** Background variables for women participating in the interviews: users of the decision aid and non-users

Characteristics of participants (*N =* 18)	Users (11)	Non-users (6)
Age (years)	23–36 (median 29)	21–39 (median 30)
Marital status		
Cohabiting	7	4
Married	4	2
Education		
Primary school or/and high school 10–12 years	3	2
College or university education 1–3 years	1	1
College or university education >3 years	7	3
Number of previous pregnancies		
Nulliparous	5	2
Multiparous	6	4
Examinations in present pregnancy		
CUB-test	5	0
CVS or AMC	0	1
Mid trimester ultrasound	11	6

### Data analysis

Data were analysed using systematic text condensation (STC) described in detail by Malterud [25]. This is a descriptive approach presenting the experiences of the participants as described by them, without the ambition of interpreting or exploring the underlying meaning of what participants say. First, all interviews were read and re-read to obtain a comprehensive image of each case. In the following step, recurrent themes reflecting the women’s experiences were identified and the text was sorted under these themes. The content in each theme was then further sorted into categories describing different aspects of the theme. Subsequently, a recontextualisation procedure was carried out to ensure that the designated themes and categories fit the unbroken original interview texts (Table 4). To achieve reliability, the second (identifying themes) and last (recontextualisation) steps of the analytical process were conducted by the researchers separately (AÅ, AS, PL and CR) and thereafter discussed together until a consensus was reached on the final themes and categories.

**Table 4:** The four steps of the analysis process in systematic text condensation according to Malterud [25]

Steps in data analysis		An example from the data
1	Read transcripts to get a total impression of the data	The impression: Women related to their previous experiences and knowledge when they talked about new knowledge and ideas gained from using the DA.
	→ Identify preliminary themes	Preliminary theme: The effect of pre-understanding
2	Identify and sort relevant text units i.e. meaning units	Text unit: *‘When we continued reading, we thought there’s not really that much you can find out about, except well, it was Down’s syndrome (…) And I don’t think that Down’s syndrome is so bad if my child should have that’.*
	→ Meaning units are sorted to create code groups	Code group: Significance of earlier experiences and knowledge
3	Condense the meaning in each code group as if it is a story told by one person	Abstraction: I understood that the test would not give us any information apart from possible DS and I don’t think that’s such a serious condition.
	→ The meaning of code is clarified through abstraction	Meaning: New knowledge about the test and women’s preconceptions affected their views.
4	Summarise the essence of each code group to a synthesis	Essence: New knowledge gained from the DA was comprehended in light of earlier understanding regarding DS.
	→ Validate the results by re-reading transcripts	Re-reading transcripts: confirmed that the code group matched statements in the interviews and that the theme recurred in most of the other interviews.

**Table 5:** Themes and categories

Themes	Categories
Appealing format	More attractive than a boring paper *Easily accessible and reliable*
New information available incorporated with earlier understanding	Selected what was important Felt supported by intelligible explanations Incorporated new facts with previous beliefs
Clarified my own standpoints	Other’s perspectives and work sheets helped Involved the partner Realised that can be difficult

## Results

Background characteristics of the participants are described in Table 3. None of the participants reported any abnormal findings from the examinations they had attended or other severe pregnancy-related complications.

Four themes emerged during the analyses (Table 5). Three themes describe women’s views and experiences of using the web-based DA: ‘Appealing format’, referring to how women perceived the format and content of the DA; ‘New information was received and incorporated with earlier understanding’, relating to how women interacted with the DA; and ‘Clarified my own standpoint’, relating to women’s increased awareness of the conscious choice it entails to take part in fetal diagnostics. The fourth theme ‘*Non*-users motives: no need for more information’ describes the motives presented by women who chose not to use the DA. The themes and categories are described below.

### Appealing format

This first theme concerns women’s opinions about significant features of the DA and what these features meant to them, specifically, being easily accessible on the Internet, more attractive than a “boring piece of paper” and being reliable.

#### Easily accessible and reliable

The women reported that the Internet was their most common source of information when they had questions on pregnancy-related issues. The fact that the women were experienced Internet users was evident in these interviews; they recognised the features of the DA from other websites they had encountered and expressed that they therefore had no problems navigating their way to the parts of the DA they were interested in.

*‘I saw right away that this is where I wanted to read every tab’.* (Participant nr 7)

The women also appreciated that the DA felt reliable, which was supported by the link to the Fetal Medicine Unit. They mentioned that they had searched on the Internet for facts related to pregnancy, but mainly ended up on chat sites, with information that could not be relied upon.

*¸It feels good … when you have a webpage that you can rely on. Of course, one can read in chat forums and places like that, but it’s not the same, and with these kinds of things, you really want it to be something from … an institution or something like that’.* (Participant nr 2)

#### More attractive than a “boring piece of paper”

The women stated that it was important to them that information was readily accessible online. They described the generic pamphlet they were given during antenatal care as being less attractive because of the lack of pictures and colours in them.

*‘It’s also easier to read the information when it is, in this way (online) than when you … just get a boring piece of paper’.* (Participant nr 11)

Some of the respondents stated that they wanted the information immediately without having to search for it; therefore, the way the website was structured was important. The women seemed aware of and sensitive to the graphic features of the website. It was especially important to them that there was not too much text on a page, the text was subdivided into sections easy to browse, and it was straightforward to navigate the site.

### New information was received and incorporated with earlier understanding

This second theme describes how women interacted with the DA, that is, how they selected what was important to them and felt supported by intelligible explanations and incorporated new facts with previous beliefs.

#### Selected what was important

The women chose the sections they felt were of value to them, and it was obvious that they knew what information they were looking for. When describing how they had used the DA, they stated that they just glanced through some parts quickly, or not at all. One woman said that although she believed she had sufficient knowledge, she still went through all the different parts to be certain she did not miss anything important. Although all the women had acquired some information on prenatal testing prior to the offer of the DA, they found the website informative.

#### Felt supported by intelligible explanations

The DA helped clarify what the tests were about, that is, not only the differences between tests, but also the implications of performing the tests. The explanation of risk figures were said to be especially valuable. The women recognised that the risk figures were difficult to understand, but when described both in words and in images, it became clearer to them what these figures meant. The women especially noted that the test results were not a ‘yes or no’ answer.

*‘It was very good information …that it is nothing you know for sure, neither if it’s high or low risk really’.* (Participant nr 2)

Also one woman, who claimed she was knowledgeable in statistics appreciated that risks were presented both as numbers and images. In addition, the woman pointed out that it was important to understand the risk figures because of the seriousness of the matter.

#### Incorporated new facts with previous beliefs

In discussing the different options, the women repeatedly returned to their own previous experiences while simultaneously referring to knowledge gained from using the DA. In their reasoning, new knowledge was incorporated into previously obtained information as well as personal values and experiences that all jointly influenced their decision.

*‘When we continued reading, we thought there’s not really that much you can find out about, except well, it was Down’s syndrome (…) And I don’t think that Down’s syndrome is so bad if my child should have that’.* (Participant nr 6)

Another woman felt that her perception of risk was overshadowed by her experiences from an earlier pregnancy, where the fetus had a serious and rare condition. She felt that her fetus could be affected regardless of any level of estimated risk and that the only test of value to her was a diagnostic test, and no additional information could have changed that standpoint.

*‘Whether I’m one out of twenty thousand or two hundred, it does not matter. My child will have Down’s syndrome regardless’.* (Participant nr 3)

Nevertheless, she commented that she probably would have appreciated the explanation on risk and hearing others’ stories had she not had these earlier experiences.

### Clarified my own standpoint

This theme described how the use of the DA affected the women’s decision-making process regarding fetal screening and diagnostics, involved their partner and thereby clarified their own standpoint.

#### Others’ perspectives and worksheets help

Participants reported that the use of the DA initiated reflections about the value of screening for fetal anomalies, and raised their awareness regarding the potential and limitations of prenatal screening examinations. Reading the expectant parents’ stories on the website, women recognised that couples in those stories based their decisions on personal values, which made women more aware of their own standpoint. These stories made them realise that there are no right or wrong approaches in these decisions, just different ones.

*‘You can interpret the figures, but to learn from other people’s stories, it is more meaningful. It’s sort of more real, (…) you can relate. One may start thinking, okay, what would we do and how would we react and what is it that we may need to talk about’.* (Participant nr 5)

Using worksheets enabled women to put their thoughts and beliefs into words. Furthermore, some women, who had made their choices before they had access to the website, appreciated that the use of this website made them more convinced that they had made the right decision.

#### Involved the partner

The women stated that the DA initiated discussions with their partner. Five of them mentioned that they reviewed the website together with their partner. The other six said that they went through it by themselves but discussed some of the issues with their partners afterwards.

*‘My partner and I went through this together, you could see if you both thought the same way, or if you had different views … For it can also be a good basis to discuss… where one stands’.* (Participant nr 8)

One woman also said that the discussion revealed differences in opinions between her and her partner, which was important for them to know.

#### Realised that it can be difficult

For some who had not reflected on the meaning of an abnormal test result, using the DA clarified the fact that prenatal tests could provide information leading to a situation where they would have to make difficult decisions. Although the women believed they knew what their standpoints were in case of abnormal findings, they realised that they could not be absolutely certain until they actually received such a result. Informants stressed the importance of raising these questions in the information to emphasise that choosing to participate entails being prepared to make a decision.

*‘We talked a lot about, whether one should do this … If you find out about this high-risk … What should one do next’.* (Participant nr 10)

### Non-users motives: no need for more information

Women who chose not to use the web-based DA when offered reported that they already had sufficient knowledge to make a decision about fetal screening and diagnostics. They either had knowledge from earlier pregnancies, or they felt that the information from the midwife or talking to friends was adequate.

*‘I believe I received the information from the midwife so, and I have so many acquaintances who are pregnant and have been. And we talk to each other and in that way, you get information’.* (Participant nr 13)

## Discussion

This qualitative study aimed to investigate pregnant women’s perceptions of the use of a web-based DA designed to initiate a process of deliberate decision-making in pregnant women concerning screening and testing for fetal anomalies. The results from the interviews showed that using the web-based DA could initiate reflections in the women about their own standpoint, as well as discussions with their partner, thus making women more aware of their partner’s views as well. Reading the expectant parents’ stories seemed especially instrumental in making women more aware of their own standpoint. Furthermore, interacting with the tool allowed them to be prepared for the results of the examinations. The DA also helped women to understand the significance of the screening result, which made them conscious that abnormal findings could involve difficult decisions. Additionally, the women appreciated that the information in the DA was brief, but clearly described, was easily accessible on the Internet and that it came from a reliable source.

When expectant parents receive the result of fetal screening for anomalies as a risk figure, counselling the expectant parents can become quite complicated, as the medical and lay conception of risk is very different. While the medical perspective refers to populations and objectively identifiable risk figures in rational terms, people judge a risk subjectively, not only by what they *think* about it but also by how they *feel* about it [26]. This implies that real risks are filtered through one’s own imagination, values and interests, which will affect expectant parents’ decision-making regarding screening tests that result in an assessment of risk for fetal anomalies. Moreover, unexpected ultrasound findings that result in a risk assessment for fetal anomaly are known to create much anxiety in expectant parents, regardless of relatively low actual levels of risk [8, 9]. Information and counselling about prenatal screening and diagnostic testing is, therefore, a challenging task for the professionals in antenatal healthcare. Because so many other things are discussed at the time of admittance to maternity care, there is a risk that information about fetal diagnostics can get lost amidst other information about pregnancy care, necessary blood tests, and medical as well as psychosocial anamnesis to assess the needs of the pregnant woman. According to the Swedish maternity care programme, there is only one planned visit with the midwife before it is time for fetal diagnostic tests; thus, there is not much time for the expectant couple to discuss and ask questions about possible procedures. In addition, no ultrasound is offered early on in pregnancy in Sweden; as a result, many couples might opt for CUB testing to be able to see the fetus on the ultrasound. As interactive decision support tools concerning screening and diagnostic testing for fetal anomalies have shown to enhance understanding of risk figures and result in more informed decisions than standard education booklets [19, 20], there are strong incentives for introducing interactive decision aid tools in maternity care.

However, lack of information or social/psychological reasons are not an optimal basis to make informed decisions about fetal diagnostic screening. A first step on the way is therefore to help pregnant women and their partners become aware that participating in prenatal screening and diagnostic testing is a personal choice, and to provide adequate support that take the lay perspectives on risk into account. Reading the ‘Expectant parents’ stories’ on the DA website clarified for some women and their partners that prenatal screening and diagnostic testing is a matter of one’s own choice.

Although it has been suggested that personal stories facilitate decision-making, they are also questioned because of lack of evidence as to whether or not these stories contribute to the effectiveness of patient decision aids [24]. Still, it is known that people use both factual information and personal stories when making healthcare decisions, although there is no clear explanation on how or why these stories influence the process of informed decision-making [24]. This study indicates that the ‘Expectant parents’ stories’ in fact were instrumental in causing women to reflect on their decision to participate in prenatal diagnostics as really being a personal choice.

Even if women have the right, according to Swedish law, to make sovereign decisions on issues concerning diagnostic testing and abortion, research has shown that expectant fathers are also involved, implying information needs of their own [27]. Men wish to be more involved in consultations before prenatal screening, not only for their own needs but also to be able to give support to their partner when dealing with difficult issues [28]. Therefore, the possibility of a DA to enhance partner involvement is important, but should be further studied with the specific aim to study men’s perceptions.

We know from the preliminary data from our on-going RCT that 40 % of the pregnant women who received a login to the website did not use it. The reason for this, as expressed by the women in this interview study, was that they believed that they had sufficient knowledge to make their decision. However, we know that expectant women do not always read the information pamphlet that they receive from the midwife [9]. Previous studies also show that some women become aware of their own knowledge gap when experiencing abnormal findings from a prenatal test [9]. In addition, it is suggested that norms and expectations from society can determine women’s choice regarding prenatal screening [29–31]; when routinely offered, prenatal screening is perceived to be as self-evident as any other pregnancy check-up and women might participate without any further considerations [32]. So the question is whether pregnant women really can make proper assessments of their needs for knowledge to support deliberate decision-making prior to the examination. If they do not read the written information and are influenced by social expectations or the perception of their peers, they risk making uninformed decisions. On the contrary, of course, it is possible that at least a subgroup of women are very well-informed and well aware of their choices and values. Therefore, further investigations are needed to examine the behaviour of pregnant women in relation to offered information or decisional aids.

The women appreciated the DA for its simple menu, giving users an immediate overview of the content of the website as well as the ability to choose to click on links for additional information, findings that are in line with those of Sawka *et al.* [33]. In addition, our findings also show that users valued facts being presented in short blocks of text and clearly explained in plain language. Moreover, the significance of an attractive design, emphasised by women in our study, has also been described earlier [34].

In evaluating the potential for the web-based tool to be implemented as part of routine care, the core elements from the diffusion of innovations theory [35] should be considered. The *relative advantage* of a web-based tool is the accessibility for the users. It is designed to be *simple* to use and anyone who is given the opportunity can *try* it out. However, what really has an impact on whether an innovation makes it or not is its *compatibility* with people’s lifestyle. Given the pervasiveness of the Internet in the lives of Swedish women who are of childbearing age, Internet-based tools seem to have a clear advantage. There are also significant advantages from the perspective of the healthcare organisation, knowing that counselling regarding prenatal testing does not always fulfil the requirements needed to support expectant parents’ informed choice [7].

### Methodological considerations

In our attempts to recruit participants for the interviews via e-mail, women either did not respond at all to the e-mail or they declined participation. Although these women previously answered questionnaires, when invited by e-mail, it is possible that participation in an interview was perceived as being more personal and therefore required a person-to-person contact with the researcher before considering participation.

Participants in this study were all Swedish-speaking and are of Northern European origin; moreover, the majority had a university education, factors that limit the transferability of our results. Therefore, the use of this web-based DA needs to be further studied with a larger group of women having more diverse backgrounds. To increase the transferability of our results, it is also important to conduct further in-depth interviews with women who chose not to use the DA.

Given that the author (AÅ) who was engaged in the development of the DA also conducted the interviews, the results could have been influenced. However, to minimise any such influence, the women did not know that the author had developed the website, and all the researchers (AÅ, CR, AS and PL) conducted the steps in the analytical process separately before working together with the results.

### Practical implications

A web-based DA has low costs, is conveniently available to a population with Internet access and can be used on one’s own terms without influence from the practitioner. The source of the website needs to be clearly stated so that users can trust that it is from an official reliable source. Moreover, it needs to be professionally designed and continually updated to reflect current practice. The web-format also allows for easy adaptation into several different languages.

### Future research

The intention of this study was not to present a complete description of all aspects of the use of a web-based decision aid. More research is needed regarding the effectiveness of the tool to enhance informed decision-making and the motivations to obtain this information as well as about parents’ information seeking behaviours when confronting prenatal screening and testing.

## Conclusions

To our knowledge, there is no previous study presenting qualitative research describing pregnant women’s perceptions on the use of an interactive web-based decision aid concerning screening and diagnostics for fetal anomalies. This research shows that a web based information and interactions with a web-based decision aid could initiate a process of deliberate decision-making regarding prenatal diagnostics in pregnant women as well as engage their partners in the decision process. Access to a web-based decision aid tool can be valuable to expectant parents in making quality decisions regarding screening for fetal anomalies and might also be a valuable resource in antenatal care. However, such a tool needs to be tested in a larger study to assess its effectiveness as well as its utility for different groups of parents.

## Abbreviations

AMC: Amniocentesis
CUB: Combined ultrasound and biochemical test
CVS: Chorionic Villus Sampling
DA: Decision aid
RCT: Randomised controlled trial
